# The chemical characteristics of different sodium iron ethylenediaminetetraacetate sources and their relative bioavailabilities for broilers fed with a conventional corn-soybean meal diet

**DOI:** 10.1186/s40104-023-00969-3

**Published:** 2024-01-30

**Authors:** Shengchen Wang, Bingxin Wu, Ling Zhu, Weiyun Zhang, Liyang Zhang, We Wu, Jiaqi Wu, Yun Hu, Tingting Li, Xiaoyan Cui, Xugang Luo

**Affiliations:** 1 College of Animal Science and Technology, Yangzhou University, Yangzhou, 225000 People’s Republic of China; 2 Mineral Nutrition Research Division, State Key Laboratory of Animal Nutrition, Institute of Animal Science, Chinese Academy of Agricultural Sciences, Beijing, 100193 China

**Keywords:** Broilers, Chelation strengths, Fe-containing enzymes, NaFeEDTA, Relative bioavailabilities

## Abstract

**Background:**

Our previous studies demonstrated that divalent organic iron (Fe) proteinate sources with higher complexation or chelation strengths as expressed by the greater quotient of formation (Q_f_) values displayed higher Fe bioavailabilities for broilers. Sodium iron ethylenediaminetetraacetate (NaFeEDTA) is a trivalent organic Fe source with the strongest chelating ligand EDTA. However, the bioavailability of Fe when administered as NaFeEDTA in broilers and other agricultural animals remains untested. Herein, the chemical characteristics of 12 NaFeEDTA products were determined. Of these, one feed grade NaFeEDTA (Q_f_ = 2.07 × 10^8^), one food grade NaFeEDTA (Q_f_ = 3.31 × 10^8^), and one Fe proteinate with an extremely strong chelation strength (Fe-Prot ES, Q_f_ value = 8,590) were selected. Their bioavailabilities relative to Fe sulfate (FeSO_4_·7H_2_O) for broilers fed with a conventional corn-soybean meal diet were evaluated during d 1 to 21 by investigating the effects of the above Fe sources and added Fe levels on the growth performance, hematological indices, Fe contents, activities and gene expressions of Fe-containing enzymes in various tissues of broilers.

**Results:**

NaFeEDTA sources varied greatly in their chemical characteristics. Plasma Fe concentration (PI), transferrin saturation (TS), liver Fe content, succinate dehydrogenase (SDH) activities in liver, heart, and kidney, catalase (CAT) activity in liver, and *SDH* mRNA expressions in liver and kidney increased linearly (*P* < 0.05) with increasing levels of Fe supplementation. However, differences among Fe sources were detected (*P* < 0.05) only for PI, liver Fe content, CAT activity in liver, SDH activities in heart and kidney, and *SDH* mRNA expressions in liver and kidney. Based on slope ratios from multiple linear regressions of the above indices on daily dietary analyzed Fe intake, the average bioavailabilities of Fe-Prot ES, feed grade NaFeEDTA, and food grade NaFeEDTA relative to the inorganic FeSO_4_·7H_2_O (100%) for broilers were 139%, 155%, and 166%, respectively.

**Conclusions:**

The bioavailabilities of organic Fe sources relative to FeSO_4_·7H_2_O were closely related to their Q_f_ values, and NaFeEDTA sources with higher Q_f_ values showed higher Fe bioavailabilities for broilers fed with a conventional corn-soybean meal diet.

## Background

As an essential trace element, iron (Fe) plays an important role in the appearance and maintenance of life on Earth [1]. Almost all living organisms require Fe to complete oxygen transport, cell proliferation and differentiation, immunity, and energy metabolism, highlighting the importance of this crucial element for physiological and biological processes [2]. Two kinds of Fe are present in food, namely, heme Fe and non-heme Fe [3]. Heme Fe can be directly absorbed by duodenal mucosal cells through endocytosis, while non-heme Fe mainly exists in the form of inorganic salts or oxides and cannot be absorbed effectively [4, 5]. The Fe ions in non-heme Fe need to be unified into Fe^2+^ under the operation of the redox system of duodenal mucosal cells, thus enabling them to enter into enterocytes via the transport of divalent metal transporter 1 [6, 7]. Moreover, Fe^2+^ entering the cells needs to be oxidized into Fe^3+^ before it can combine with transferrin and be utilized by the whole body through the blood circulation [8]. Therefore, selecting a high-quality dietary Fe source is especially important for animals to ensure its absorption, bioavailability, metabolic homeostasis, and normal growth.

For broiler chickens, traditionally, the Fe additives in diets are mainly inorganic salts. However, because of the shortcomings of inorganic Fe sources, such as low bioavailability, high hygroscopicity and vulnerability to destruction by other nutrients in diets, organic Fe sources have attracted increasing attention and gradually been developed and utilized as a substitute for inorganic Fe sources [9, 10]. Currently, a number of evidences have shown that under the protection of the ligand, the Fe ions in organic Fe sources could resist precipitation or adsorption of various inhibitors in the digestive tract, thus being better absorbed and utilized by intestinal epithelial cells [11]. Of note, in our previous studies, we have demonstrated that the relative bioavailabilities of divalent organic Fe sources in broilers are closely related to their complexation or chelation strengths as expressed by the quotient of formation (Q_f_) value (a quantitative measurement of complex or chelation strength) [12], and organic Fe proteinate sources with greater Q_f_ values display higher Fe absorption and bioavailabilities [9, 10, 13–16]. Sodium iron ethylenediaminetetraacetate (NaFeEDTA) is a trivalent organic Fe source with the strongest chelating ligand EDTA. It has been widely used as a Fe supplement in human foods because it offers certain advantages, such as no irritation of the stomach and gut as well as high Fe absorption as it avoids interferences from phytic acid and other anti-nutritional factors in the gut [17–19]. However, the absorption and bioavailability of Fe when administered as NaFeEDTA, and whether NaFeEDTA is a suitable additive to diets as a dietary Fe supplement for broilers and other agricultural animals remain unexplored to date. Therefore, we hypothesized that the NaFeEDTA with the highest Q_f_ value would be better than the inorganic Fe sulfate (FeSO_4_∙7H_2_O) and the organic Fe proteinate with an extremely strong chelation strength (Fe-Prot ES, Q_f_ value = 8,590, [12]) in improving Fe bioavailability in broilers.

To test the above hypothesis, in the present study, we analyzed the chemical characteristics of different NaFeEDTA sources and investigated the effects of different Fe sources and added Fe levels on the growth performance, hematological indices, Fe contents, activities, and gene expressions of Fe-containing enzymes in various tissues of broilers so as to evaluate the bioavailabilities of different NaFeEDTA sources and Fe-Prot ES relative to FeSO_4_∙7H_2_O for broilers fed with a conventional corn-soybean meal diet from 1 to 21 days of age.

## Methods

### Fe sources

A total of 12 commercial NaFeEDTA products (4 feed grade and 8 food grade), 1 feed grade Fe-Prot ES, and 1 reagent grade FeSO_4_∙7H_2_O were purchased from several manufacturers or independent distributors.

### Analysis of Fe contents in NaFeEDTA products

The total Fe contents in NaFeEDTA sources were analyzed by using the sodium thiosulfate (Na_2_S_2_O_3_) titration method [20]. In brief, approximately 0.5 g of each NaFeEDTA product was weighed and added into an iodine flask; 40 mL of deionized water, 3.0 g of potassium iodide, and 20 mL of hydrochloric acid were added into the flask, and the mixture was shaken well and left to settle in the dark for about 5 min. Then, the mixed liquid was titrated with Na_2_S_2_O_3_ standard solution until the color of the solution changed to blue, and 2.0 mL of starch indicator solution was immediately added into the solution until the blue color disappeared. The total consumed volume of Na_2_S_2_O_3_ standard solution was V1 (mL). A blank solution with no sample was again titrated with Na_2_S_2_O_3_ according to the method described above, and the consumed volume V2 (mL) of Na_2_S_2_O_3_ standard solution was also recorded. The Fe content (W1) in NaFeEDTA was calculated using the following formula: $\textrm{W}2=\frac{\frac{\textrm{V}1-\textrm{V}2}{100}\times \textrm{C}1\times \textrm{M}1}{\textrm{M}1}$ (C1 = 0.1005 mol/L is the molar concentration of Na_2_S_2_O_3_, and M1 = 55.8 g/mol is the molar mass of Fe). The average of the results from duplicate determinations was taken as the analyzed result of the Fe content in each NaFeEDTA source.

### Determinations of EDTA, moisture, lead, and arsenic contents, solubilities, dissolution rates, and Q_f_ values in NaFeEDTA sources

NaFeEDTA sources of which the Fe contents meet the national standard (12.5%–13.5% Fe contents) of food grade NaFeEDTA were selected for subsequent analyses of chemical characteristics [20]. The total EDTA content in each NaFeEDTA source was analyzed using the calcium acetate monohydrate (Ca(CH_3_COO)_2_·H_2_O) titration method [20]. In brief, approximately 0.8 g of each NaFeEDTA product was weighed and dissolved with 75 mL of ultrapure water in a 250 mL volumetric flask. Triethanolamine and sodium hydroxide solutions were added into the flask and the sample solution pH was adjusted to 12.5–13.0. Subsequently, 30 mg of hydroxynaphthol blue indicator was added into the volumetric flask and the mixed liquid was titrated with a Ca(CH_3_COO)_2_·H_2_O solution until the color of the solution changed from blue to red. The volume of the consumed Ca(CH_3_COO)_2_·H_2_O solution was V2, and the EDTA content (W2) was calculated using the following formula: $\textrm{W}2=\frac{\textrm{V}2\times \textrm{C}2\times \textrm{M}2}{1,000\times \textrm{M}2}$ (C2 = 0.25 mol/L is the molar concentration of Ca(CH_3_COO)_2_·H_2_O, and M2 = 292.24 g/mol is the molar mass of EDTA). The moisture content of NaFeEDTA was analyzed using the drying method as described by Meng et al. [21]. The lead and arsenic contents were analyzed by using AOAC’s wet digestion method as described by the determination of multiple elements in National Food Safety Standards of China [22]. The Fe dissolution rate of Fe in NaFeEDTA in deionized H_2_O and the solubilities of NaFeEDTA in deionized H_2_O, 0.2 mol/L HCl-KCl buffer (pH 2.0), or 0.1 mol/L K_2_HPO_4_-KH_2_PO_4_ buffer (pH 5.0) were analyzed as described by Zhang et al. [12]. The average of the results from duplicate determinations was taken as the analyzed results of the EDTA content, moisture content, lead and arsenic contents, solubility, and dissolution rate of each NaFeEDTA source.

Finally, one feed grade NaFeEDTA and one food grade NaFeEDTA with similar total Fe contents, Fe dissolution rates, and solubilities were selected for the determination of Q_f_ values. The Q_f_ value of each NaFeEDTA source was determined using polarography with a hanging mercury drop electrode according to a previously described method [12]. The NaFeEDTA product was dissolved in deionized water to prepare a saturated NaFeEDTA solution with a Fe concentration of approximately 0.1 mol/L. Subsequently, the NaFeEDTA solution was diluted with 0.1 mol/L potassium sodium tartrate-ethylenediamine buffer solution (pH 12.0) to prepare of the final solution containing 1 × 10^−3^ mol/L Fe for anaerobic electrochemical measurements with a N purge. Meanwhile, a FeCl_3_·6H_2_O solution containing 1 × 10^−3^ mol/L Fe was prepared and used as the control. The half-wave potential (E_1/2_) of either the NaFeEDTA solution or the FeCl_3_·6H_2_O solution was measured by the 844 Professional VA (Metrohm Herisau, Switzerland). Their shift in half-wave potential (ΔE_1/2_) was used to calculate the Q_f_ value as described by Zhang et al. [12]. The average of the results from triplicate determinations was taken as the analyzed result of the Q_f_ value for each NaFeEDTA source. The results indicated that the Q_f_ values of feed grade NaFeEDTA and food grade NaFeEDTA were 2.07 × 10^8^ and 3.31 × 10^8^, respectively.

### Animals, diets, and experimental design

A total of 728 1-day-old Arbor Acres (AA) commercial male broiler chicks were randomly allotted by bodyweight to 1 of 13 treatments with 7 replicate cages (8 birds/cage). A completely randomized design was employed involving a 1 (control) + 4 (Fe sources) × 3 (added Fe levels) factorial arrangement of treatments. Birds were fed a Fe-unsupplemented corn-soybean meal basal diet (control, containing 67.90 mg of Fe/kg by analysis), and a basal diet supplemented with 20, 40, or 60 mg of Fe/kg from 1 of 4 Fe sources [reagent grade FeSO_4_∙7H_2_O, feed grade Fe-Prot ES (Q_f_ value = 8,590), feed grade NaFeEDTA (Q_f_ value = 2.07 × 10^8^), or food grade NaFeEDTA (Q_f_ value = 3.31 × 10^8^)] for a duration of 21 d. All chicks were kept in electrically heated, thermostatically controlled, stainless-steel cages (90 cm × 70 cm × 45 cm) with waterers and feeders for 21 d. During this period, chicks were maintained at a 24 h-constant lighting and had free access to experimental diets and tap water (with no detectable Fe). Dead chicks were daily recorded, and bodyweight as well as feed intake of chicks per cage were measured at the beginning of the experiment and at 21 days of age to calculate the average daily gain (ADG), average daily feed intake (ADFI), and feed to gain ratio (F/G) of birds from 1 to 21 days of age.

The basal corn-soybean meal diet (Table 1, containing 67.90 mg of Fe/kg by analysis) was formulated to meet or exceed the requirements of all other nutrients except for Fe for starter broilers, according to the recommendation of the Chinese Feeding Standard of Chicken [23]. According to the experimental treatments described above, the 4 Fe sources were added to the basal diet, respectively. The FeSO_4_∙7H_2_O was reagent grade (purity > 99%, 19.5% Fe by analysis), and the Fe-Prot ES was feed grade [10.16% Fe, 57.86% total amino acids (3.18% Lys, 0.68% Met, 0.28% Cys, 7.69% Asp, 2.35% Ser, 5.30% Glu, 0.70% Thr, 13.72% Gly, 1.51% Arg, 4.0% Ala, 8.80% Pro, 1.25% Val, 1.20% Phe, 0.60% Ile, 1.51% Leu, and 5.09% His), Q_f_ value = 8,590 by analysis]. Both the FeSO_4_∙7H_2_O and the Fe-Prot ES were the same as in our previous study [12]. The feed grade NaFeEDTA (13.14% Fe, Q_f_ value = 2.07 × 10^8^ by analysis) and the food grade NaFeEDTA (13.14% Fe, Q_f_ value = 3.31 × 10^8^ by analysis) were the latest selected trivalent organic Fe sources with super extremely strong chelation strength. In addition, variable small amounts of L-lysine monohydrochloride or DL-methionine were added to respective experimental diets to balance the levels of lysine and methionine in all of 13 treatment diets. The analyzed Fe contents of diets are listed in Table 2. Diets were fed to birds in the mash form.

**Table 1:** Composition of the basal diet for 1- to 21-day-old broilers

Items	Contents
Ingredients, %	
Corn	53.69
Soybean meal	37.39
Soybean oil	4.83
CaHPO_4_^1^	1.86
CaCO_3_^1^	1.17
Sodium chloride^1^	0.30
DL-methionine^2^	0.32
Micronutrients^3^	0.29
Cornstarch^4^	0.15
Nutrient levels	
ME, kcal/kg	3,027
Crude protein^5^, %	21.62
Lysine, %	1.12
Methionine, %	0.61
Methionine + cysteine, %	0.91
Ca^5^, %	1.02
Nonphytate P, %	0.45
Fe^5^, mg/kg	67.90

^1^Reagent grade

^2^Feed grade

^3^Provided per kilogram of diet: vitamin A (as all-*trans* retinol acetate), 12,000 IU; vitamin D_3_, 4,500 IU; vitamin E (as DL-α-tocopheryl acetate), 33 IU; vitamin K_3_, 3 mg; vitamin B_1_, 3 mg; vitamin B_2_, 9.6 mg; vitamin B_6_, 4.5 mg; vitamin B_12_, 0.03 mg; calcium pantothenate, 15 mg; niacin, 54 mg; folic acid, 1.5 mg; biotin, 0.15 mg; choline, 700 mg; Cu (CuSO_4_∙5H_2_O), 6 mg, Zn (ZnSO_4_∙7H_2_O), 60 mg; Mn (MnSO_4_∙H_2_O), 110 mg; I (Ca(IO_3_)_2_·H_2_O), 0.35 mg; Se (Na_2_SeO_3_), 0.35 mg

^4^The Fe additives, L-lysine HCl or DL-methionine were added to diets by replacing an equal weight of cornstarch

^5^Analyzed values. Each value is based on triplicate determinations

**Table 2:** Analyzed Fe contents in diets for 1- to 21-day-old broilers

Fe source^1^	Added Fe, mg/kg	Analyzed Fe^2^, mg/kg (as-fed basis)
Control	0	67.9
FeSO_4_∙7H_2_O	20	86.6
	40	112.2
	60	132.0
Fe-Prot ES	20	85.9
	40	106.9
	60	128.3
Feed grade NaFeEDTA	20	85.4
	40	106.1
	60	128.6
Food grade NaFeEDTA	20	83.30
	40	104.45
	60	123.77

^1^Fe-Prot ES represents the iron proteinate with extremely strong chelation strength (Q_f_ = 8,590). Feed grade NaFeEDTA represents the sodium iron ethylenediaminetetraacetate with super extremely strong chelation strength (Q_f_ = 2.07 × 10^8^) at feed grade. Food grade NaFeEDTA represents the sodium iron ethylenediaminetetraacetate with super extremely strong chelation strength (Q_f_ = 3.31 × 10^8^) at food grade. The same as below

^2^Values of analyzed Fe contents are based on triplicate determinations of diets and reported on an as-fed basis

### Sample collections and preparations

Before initiation of the trial, samples of feed ingredients and diets from 13 treatment groups were taken for analyses of dietary crude protein, Fe, and calcium (Ca) contents, and the tap water was collected to analyze the Fe content. At 21 days of age, 3 chicks were selected from each cage according to the mean bodyweight within the cage after a 12-h fast. Samples of blood were obtained from 3 birds through wing vein puncture. Part of these blood samples was immediately used to measure hemoglobin (Hb) concentration and hematocrit (Hct), and another part was centrifuged (3,000 × *g*, at 4 °C) for 15 min to harvest plasma and then stored at −20 °C until analyses of plasma Fe concentration (PI) and total Fe binding capacity (TIBC). Subsequently, these chicks were sacrificed to collect heart, liver, kidney, spleen, pancreas, and left tibia samples. Part of samples was stored at −20 °C to determine tissue Fe contents and activities of succinate dehydrogenase (SDH), catalase (CAT), and cytochrome c oxidase (COX). And another part was deep-frozen in liquid nitrogen and stored at −80 °C to detect *SDH*, *CAT*, and *COX* gene expression levels. Before analyses, the samples from 3 individual birds were pooled into 1 sample based on replicate cage, and thus each treatment had a total of 7 replicate samples.

### Sample analyses

#### Determinations of Fe, Ca, and dietary crude protein contents

After wet digestions with HNO_3_ and HCIO_4_, Fe contents in feed ingredient, diet, water, and tissue samples as well as Ca contents in feed ingredient and diet samples were measured by the 5110 inductively coupled plasma optical emission spectrometry (Agilent Technologies Australia (M) Pty Ltd., Australia) as described previously [12, 24]. Yellow soybean powder (GBW 10013 (GSB-4), National Research Center of Standard Materials, Beijing, China) and pork liver powder (GBW 10051 (GSB-29), National Research Center of Standard Materials, Beijing, China) were used as standard references to validate the analyses of Fe and Ca contents. The crude protein contents in feed ingredient and diet samples were determined according to the methods of the Association of Official Analytical Chemists [25].

#### Determinations of hematological indices and activities of Fe-containing enzymes in tissues

Hematological indices measured in the present study included Hb concentration, Hct, PI, TIBC, and transferrin saturation (TS), while activities of Fe-containing enzymes included CAT, SDH, and COX activities. The Hb concentration and Hct in fresh blood samples were determined by using automatic blood chemistry analyzer (MEK-8222 K, Optoelectronics). The PI and TIBC in blood plasma and activities of Fe-containing enzymes (CAT and SDH) in heart, liver, and kidney were measured using a microplate reader with commercial chemical testing kits according to the manufacturer’s instructions (Nanjing Jiancheng Bioengineering Institute, Nanjing, China). The activity of COX in liver was determined by using an ELISA kit provided by Qiaodu Biotechnology Company, Shanghai, China. The TS in plasma was calculated according to the following equation: TS (%) = (PI/TIBC) × 100%, and the protein concentrations in tissue supernatants were measured using a bicinchoninic acid (BCA) Protein Assay kit (catalog number 23225; Thermo Scientific, Rockford, IL, USA).

#### Total RNA isolation and quantitative real-time polymerase chain reaction (RT-qPCR)

Total RNA was isolated from heart, liver, and kidney tissues using the Trizol reagent (Invitrogen, Thermo Fisher Scientific), and the concentration and purity of RNA were estimated by using a spectrophotometer at 260/280 nm. First-strand cDNA for RT-qPCR was obtained using the SuperScript III First-Strand Synthesis for RT-PCR kit (cat No. 18080–051, Invitrogen). After formulating the premix system diluted cDNA (1 μL), 2× SYBR Green PCR Master Mix (5 μL), ROX reference (0.2 μL), PCR-grade water (3.4 μL), and each primer (0.2 μL, 10 μmol/L), the quantification of mRNA expression was performed using the ABI PRISM 7500 Sequence Detection System (Applied Biosystems, Foster City, CA, USA). The primers of *CAT*, *SDH*, *COX*, β-actin, and glyceraldehyde-3-phosphate dehydrogenase (*GAPDH*) mRNA were synthesized by Generay Biotech (Shanghai, China) and are listed in Table 3. β-actin and *GAPDH* genes were used as internal reference genes to complete data normalization according to the 2^-ΔΔCt^ method.

**Table 3:** Gene-special primers used in the real-time quantitative reverse transcription PCR

Genes^1^	GenBank ID^2^	Primer sequences	Length, bp
*CAT*	NM_0010311215.2	F:5′-TTGCTGGAGAATCTGGGTC-3′	186
		R:5′-CCTTCAAATGAGTCTGAGGGTT-3′	
*SDH*	NM_001080875.2	F:5′-TACAAATCCATCGAGCCTTAC-3′	111
		R:5′-GCACTCATAGAGTCCGTCCA-3′	
*COX*	JX_16009.1	F:5′-GCAGGTGTCGGTCAAGT-3′	187
		R:5′-GGTTGCGGTCGGTAACTAA-3′	
β-actin	NM_205518.1	F:5′-CGGTACCAATTACTGGGTGTTTAGATG-3′	163
		R:5′-GCCTTCATTCATTCACATCTATCACTGG-3′	
*GAPDH*	NM_204305.1	F:5′-CTTTGGCATTGTGGAGGGTC-3′	128
		R:5′-ACGCTGGGATGATGTTCTGG-3′	

^1^*CAT* Catalase, *SDH* Succinate dehydrogenase, *COX* Cytochrome C oxidase, *GAPDH* Glyceraldehyde-3-phosphate dehydrogenase

#### Protein extraction and Western blotting

Total protein was obtained from 50 mg of frozen liver samples according to the method previously described [26], and the concentration of extracted protein was determined via a BCA assay kit. Subsequently, each protein sample (40 or 60 μg/group) was loaded and separated on 12% SDS-PAGE gels, blocked with 5% nonfat milk-TBST for 2 h at 37 °C, and incubated with primary antibodies CAT (Cat. No. A11780, ABclonal, 1:1,000), SDH (Cat. No. A10821, ABclonal, 1:1,000), COX (Cat. No. A7531, ABclonal, 1:1,000), and GAPDH (Cat. No. AC001, ABclonal, 1:1,000). On the second day, the membranes were washed with TBST and incubated with HRP-Goat Anti-Rabbit IgG secondary antibody (Cat. No. HX2031, Huaxingbio, 1:5,000). Finally, the bands were visualized on a chemiluminescence image scanner (Tanon). The ratio of CAT, SDH, or COX protein band intensity to the internal reference GAPDH protein band intensity was used to reflect the protein expression level.

### Statistical analyses

Single degree of freedom contrast was used to test the differences of the data between all supplemental Fe treatments and the control, thus indicating the effect of Fe supplementation [27]. Data excluding the control group were further analyzed as a 4 × 3 (Fe source × added level) factorial arrangement of treatments by two-way ANOVA via the GLM procedure in SAS 9.4 (SAS Institute Inc., Cary, NC, USA). The statistical model included Fe source, added Fe level and their interaction. Orthogonal comparisons for the linear response of dependent variables to independent variables were used to establish the inferences about one (added Fe level) of the main effects. The relative bioavailability values of feed grade Fe-Prot ES, feed grade NaFeEDTA, and food grade NaFeEDTA were determined using FeSO_4_∙7H_2_O as standard source by slope ratio comparison from multiple linear regressions [28]. Moreover, the daily dietary Fe intake (based on Fe assays of diets) rather than added Fe levels was used as the independent variable to calculate the regressions. Slope ratios and their SE were assessed using the error propagation method as described by Littell et al. [29]. Differences between Fe sources were identified by differences in their respective regression coefficients. The replicate cage was regarded as the experimental unit, and the level of statistical significance was set at *P* <  0.05.

## Results

### Chemical characteristics of different NaFeEDTA sources

The results were shown in Table 4. Total Fe contents varied considerably across the 12 tested NaFeEDTA sources, ranging from the least of 2.58% in feed grade NaFeEDTA 3 to the highest of 13.33% in food grade NaFeEDTA 1. Only 5 NaFeEDTA products (2 feed grade and 3 food grade) had Fe contents that were within the 12.5%–13.5% range as specified by the national standard of food grade NaFeEDTA [20]. Further analyses of these 5 Fe products showed that the EDTA contents ranged from 68.50% to 70.16%; the molar ratios of EDTA and Fe were about 1:1; the range of moisture contents was 7.80%–8.59%, and heavy metals lead and arsenic contents remained 0.20 mg/kg. Meanwhile, the Fe dissolution rates of the 5 NaFeEDTA products in deionized H_2_O ranged from 68.26% to 83.05%, and their solubilities in deionized H_2_O and two buffers were high, ranging from 89.95% to 99.05%. Finally, the feed grade NaFeEDTA 2 and the food grade NaFeEDTA 4 were chosen for Q_f_ value detection as they had the same Fe content as well as similar Fe dissolution rates and solubilities. The Q_f_ values of the feed grade NaFeEDTA 2 and the food grade NaFeEDTA 4 were 2.07 × 10^8^ and 3.31 × 10^8^, respectively, indicating that the two NaFeEDTA sources had super extremely strong chelation strengths. Therefore, these two NaFeEDTA sources were used in the subsequent in vivo broiler experiment.

**Table 4:** Chemical characteristics of different NaFeEDTA sources

Product No.	Total Fe^1^, %	EDTA^1^, %	Molar ratio, mole:mole^1,3^	Q_f_ value^2,4^	Moisture content, %	Lead^1^, mg/kg	Arsenic^1^, mg/kg	Fe dissolution rate^1^, %	Solubility^1^, %		
									Deionized H_2_O	HCl-KCl buffer (pH 2.0)	KH_2_PO4-K_2_HPO_4_ buffer (pH 5.0)
Feed grade NaFeEDTA 1	13.15	70.16	1.02:1	–	7.86	0.07	0.013	68.26	96.61	91.54	91.26
Feed grade NaFeEDTA 2	13.14	69.27	1.01:1	2.07 × 10^8^	7.73	0.1	0.065	81.13	97.50	92.04	92.23
Feed grade NaFeEDTA 3	2.58	–	–	–	–	–	–	–	–	–	–
Feed grade NaFeEDTA 4	6.29	–	–	–	–	–	–	–	–	–	–
Food grade NaFeEDTA 1	13.33	69.43	1:1	–	8.56	0.08	0.076	70.71	94.32	90.75	89.95
Food grade NaFeEDTA 2	6.70	–	–	–	–	–	–	–	–	–	–
Food grade NaFeEDTA 3	6.27	–	–	–	–	–	–	–	–	–	–
Food grade NaFeEDTA 4	13.14	68.50	1:1	3.31 × 10^8^	7.82	0.12	0.043	83.05	98.11	92.2	91.3
Food grade NaFeEDTA 5	12.93	68.60	1.01:1	–	8.52	0.01	0.11	79.88	99.05	94.44	93.41
Food grade NaFeEDTA 6	6.56	–	–	–	–	–	–	–	–	–	–
Food grade NaFeEDTA 7	6.64	–	–	–	–	–	–	–	–	–	–
Food grade NaFeEDTA 8	12.18	–	–	–	–	–	–	–	–	–	–

^1^Each value based on duplicate measurements

^2^Each value based on triplicate measurements

^3^The molar ratios were calculated according to the equation: molar ratio = (the total EDTA concentration/the molecular weight of EDTA)/(the Fe concentration/the atomic weight of Fe)

^4^*Q*_*f*_ quotient of formation, which is used to describe the complex or chelation strength of organic trace elements

### Growth performance

Compared to the control, dietary Fe supplementation had no effect (*P* > 0.05) on ADG, ADFI, and F:G from 1 to 21 days of age; furthermore, Fe source, added Fe level, and their interaction did not affect (*P* > 0.47) all of the above indicators as described in our previous study [30].

### Hematological indices

The results were shown in Table 5. Compared to the control, dietary Fe supplementation had no effect (*P* > 0.05) on Hb concentration, Hct and TIBC, but significantly increased (*P* < 0.05) PI and TS. The Hb concentration, Hct, PI, TIBC, and TS were not affected (*P* > 0.09) by the Fe source and the interaction between Fe source and added Fe level, but PI and TS were affected (*P* < 0.0001) by the added Fe level, and increased linearly (*P* < 0.0001) with increasing dietary Fe levels.

**Table 5:** Effects of dietary Fe on hematological indices of broilers on d 21

Fe source	Added Fe, mg/kg	Hb, g/L	Hct, L/L	PI, μg/mL	TIBC, μg/mL	TS, %
Control^1^	0	78.1	30.7	1.05*	3.62	30.1*
FeSO_4_∙7H_2_O ^1^	20	76.8	29.8	1.10	3.19	34.2
	40	78.1	30.6	1.23	3.58	36.0
	60	79.9	31.1	1.30	3.23	41.8
Fe-Prot ES^1^	20	81.5	31.9	1.03	3.57	28.5
	40	80.7	31.2	1.15	3.22	38.3
	60	79.4	31.0	1.34	3.42	40.5
Feed grade NaFeEDTA^1^	20	78.5	31.2	1.09	3.40	32.9
	40	80.5	31.3	1.20	3.18	37.6
	60	78.2	30.3	1.44	3.49	42.9
Food grade NaFeEDTA^1^	20	77.0	30.0	1.14	3.68	31.3
	40	78.6	30.9	1.27	3.37	38.2
	60	80.7	31.6	1.49	3.40	45.3
Pooled SE		0.94	0.37	0.06	0.29	2.50
Fe source^2^	FeSO_4_∙7H_2_O	78.3	30.6	1.21	3.33	37.3
	Fe-Prot ES	80.5	31.4	1.18	3.40	35.8
	Feed grade NaFeEDTA	79.1	30.9	1.24	3.36	37.8
	Food grade NaFeEDTA	78.8	30.8	1.30	3.48	38.3
Pooled SE		0.82	0.32	0.04	0.18	1.57
Added Fe level^3^, mg/kg	20	78.5	30.7	1.09^c^	3.46	31.7^c^
	40	79.4	31.0	1.21^b^	3.34	37.5^b^
	60	79.6	31.1	1.39^a^	3.39	42.6^a^
Pooled SE		1.63	0.64	0.03	0.16	1.33
*P*-value	Fe source	0.3894	0.4786	0.0935	0.9434	0.7114
	Added Fe level	0.5756	0.7336	< 0.0001	0.8595	< 0.0001
	Fe Source × added Fe level	0.5201	0.2347	0.8045	0.8857	0.7945
	Linear effect^4^	–	–	< 0.0001	–	< 0.0001

*Hb* Hemoglobin, *Hct* Hematocrit, *PI* Plasma iron, *TIBC* Total iron binding capacity, *TS* Transferrin saturation

^1^Data represent the means of 7 replicate cages (*n* = 7)

^2^Data represent the means of 21 replicate cages (*n* = 21)

^3^Data represent the means of 28 replicate cages (*n* = 28)

^4^Linear effects of added Fe levels

^a–c^Means with different superscripts within the same column differ (*P* < 0.05)

*Different (*P* < 0.05) from all Fe supplemental groups

### Fe contents in tissues

The data were shown in Table 6. Compared to the control, dietary Fe addition had no effect (*P* > 0.05) on Fe contents in heart and tibia ash, but increased (*P* < 0.05) Fe contents in kidney and liver. The Fe contents in kidney and tibia ash were not affected (*P* > 0.05) by the Fe source, added Fe level, and their interaction. The Fe contents in heart and liver were affected (*P* < 0.01) by the Fe source. Chicks that received the diet supplemented with either feed grade NaFeEDTA or food grade NaFeEDTA had higher (*P* < 0.04) heart Fe contents than those fed with the diet supplemented with FeSO_4_∙7H_2_O; heart Fe contents were higher (*P* = 0.0065) for food grade NaFeEDTA than for Fe-Prot ES with no differences (*P* > 0.08) between FeSO_4_∙7H_2_O and Fe-Prot ES, Fe-Prot ES and feed grade NaFeEDTA, as well as feed grade NaFeEDTA and food grade NaFeEDTA. Compared to the chicks fed with the diet supplemented with FeSO_4_∙7H_2_O, the chicks fed the diets supplemented with the 3 organic Fe sources had increased (*P* < 0.04) Fe contents in liver, and no differences (*P* > 0.32) were observed among the 3 organic Fe sources. In addition, liver Fe content was affected (*P* = 0.0348) by added Fe level, and increased linearly (*P* < 0.0001) with increasing added Fe levels. Heart Fe content was not affected (*P* > 0.25) by added Fe level and the interaction between Fe source and added Fe level, and did not increase linearly (*P* > 0.06) with increasing added Fe levels.

**Table 6:** Effects of dietary Fe on Fe contents in tissues of broilers on d 21

Fe source	Added Fe, mg/kg	Heart Fe, μg/g (fresh basis)	Kidney Fe, μg/g (fresh basis)	Liver Fe, μg/g (fresh basis)	Tibia ash Fe, μg/g (ash basis)
Control^1^	0	32.6	28.0*	75.3*	243
FeSO_4_∙7H_2_O^1^	20	30.6	39.1	97.7	252
	40	33.2	47.3	101	240
	60	35.7	48.8	102	247
Fe-Prot ES^1^	20	31.9	50.8	106	255
	40	32.7	57.4	117	253
	60	36.4	53.2	121	264
Feed grade NaFeEDTA^1^	20	36.9	42.6	105	260
	40	35.6	53.0	123	246
	60	35.3	50.9	133	258
Food grade NaFeEDTA^1^	20	37.1	49.3	116	250
	40	38.2	46.3	118	251
	60	36.6	48.6	134	259
Pooled SE		1.58	3.67	8.06	7.24
Fe source^2^	FeSO_4_∙7H_2_O	33.2^C^	45.1	100^B^	246
	Fe-Prot ES	34.1^BC^	53.8	115^A^	257
	Feed grade NaFeEDTA	35.9^AB^	48.8	120^A^	255
	Food grade NaFeEDTA	37.3^A^	48.1	122^A^	253
Pooled SE		0.91	2.06	4.78	4.36
Added Fe level^3^, mg/kg	20	34.1	45.5	106^b^	254
	40	35.2	51.0	115^ab^	247
	60	36.0	50.3	123^a^	257
Pooled SE		0.79	1.82	4.31	3.83
*P*-value	Fe source	0.0058	0.0532	0.0098	0.3819
	Added Fe level	0.2659	0.0782	0.0348	0.2117
	Fe Source × added Fe level	0.2505	0.5518	0.8321	0.9325
	Linear effect^4^	0.0634	–	< 0.0001	–

^1^Data represent the means of 7 replicate cages (*n* = 7)

^2^Data represent the means of 21 replicate cages (*n* = 21)

^3^Data represent the means of 28 replicate cages (*n* = 28)

^4^Linear effects of added Fe levels

^A–C^Means with different superscripts between Fe sources differ (*P* < 0.05)

^a,b^Means with different superscripts between added Fe levels differ (*P* < 0.05)

*Different (*P* < 0.05) from all Fe supplemental groups

### Activities of Fe-containing enzymes in tissues

The data were shown in Table 7. There were no differences (*P* > 0.05) in CAT activities in heart and kidney between the chicks fed with the control diet and those fed with all Fe-supplemented diets. The Fe source, added Fe level, and their interaction did not affect (*P* > 0.35) CAT activities in these two tissues. However, compared to the control, dietary Fe supplementation increased (*P* < 0.05) CAT, SDH, and COX activities in liver, and SDH activities in heart and kidney. Both liver CAT activity and kidney SDH activity were affected (*P* < 0.006) by Fe source. Compared to the FeSO_4_∙7H_2_O, the 3 organic Fe sources increased (*P* < 0.01) liver CAT activities, and either feed grade NaFeEDTA or food grade NaFeEDTA increased (*P* < 0.003) kidney SDH activities with no differences (*P* > 0.08) among the 3 organic Fe sources. No difference (*P* = 0.1631) was observed in kidney SDH activity between FeSO_4_∙7H_2_O and Fe-Prot ES either. The CAT, SDH and COX activities in liver, and SDH activities in heart and kidney were affected (*P* < 0.05) by the added Fe level, and increased linearly (*P* < 0.003) as the increase of added Fe levels. However, no effects (*P* > 0.16) were found regarding the interaction between Fe source and added Fe level on the above 5 indices and Fe source on SDH activities in heart and liver and liver COX activity.

**Table 7:** Effects of dietary Fe on activities of tissue Fe-containing enzymes of broilers on d 21

Fe source	Added Fe, mg/kg	CAT activity, U/mg protein			SDH activity, U/mg protein			COX activity, mU/mg protein
		Heart	Kidney	Liver	Heart	Kidney	Liver	Liver
Control^1^	0	2.21	19.0	16.2*	3.44*	4.34*	2.33*	28.7*
FeSO_4_∙7H_2_O^1^	20	2.23	22.9	16.5	3.57	4.43	2.37	30.1
	40	2.13	20.1	16.6	3.96	4.81	2.57	31.6
	60	2.19	20.6	16.7	4.12	4.92	2.81	34.9
Fe-Prot ES^1^	20	2.07	20.6	16.9	3.63	4.72	2.43	29.9
	40	2.25	20.8	17.4	3.99	5.28	2.69	34.5
	60	2.07	18.6	18.2	4.41	5.45	2.91	35.5
Feed grade NaFeEDTA^1^	20	2.02	19.5	16.7	3.95	5.32	2.60	29.1
	40	2.08	20.5	17.4	4.40	5.51	2.78	34.5
	60	2.15	19.4	18.4	4.62	6.22	2.82	35.3
Food grade NaFeEDTA^1^	20	2.08	18.5	17.4	4.19	4.98	2.64	31.1
	40	1.92	18.9	17.9	4.47	5.58	2.79	33.7
	60	2.00	20.2	19.0	4.65	6.46	2.86	36.0
Pooled SE		0.18	1.40	0.41	0.31	0.36	0.17	1.71
Fe source^2^	FeSO_4_∙7H_2_O	2.18	21.2	16.6^B^	3.88	4.72^B^	2.58	32.2
	Fe-Prot ES	2.13	19.8	17.5^A^	4.00	5.15^AB^	2.68	33.3
	Feed grade NaFeEDTA	2.08	19.8	17.5^A^	4.32	5.68^A^	2.73	33.0
	Food grade NaFeEDTA	2.00	19.2	18.1^A^	4.44	5.67^A^	2.76	33.6
Pooled SE		0.10	0.81	0.24	0.19	0.21	0.10	1.01
Added Fe level^3^, mg/kg	20	2.10	20.4	16.9^b^	3.83^b^	4.86^b^	2.51^b^	30.1^b^
	40	2.09	19.9	17.3^b^	4.20^ab^	5.29^ab^	2.71^ab^	33.6^a^
	60	2.10	19.7	18.1^a^	4.45^a^	5.76^a^	2.85^a^	35.4^a^
Pooled SE		0.09	0.70	0.21	0.17	0.18	0.09	0.86
*P*-value	Fe source	0.6307	0.3541	0.0006	0.1672	0.0052	0.5973	0.7885
	Added Fe level	0.9974	0.7886	0.0011	0.0480	0.0049	0.0244	0.0002
	Fe Source × added Fe level	0.9529	0.6721	0.7452	0.9993	0.8438	0.9812	0.9456
	Linear effect^4^	–	–	0.0002	0.0029	0.0006	0.0020	< 0.0001

*CAT* Catalase, *SDH* Succinate dehydrogenase, *COX* Cytochrome c oxidase

^1^Data represent the means of 7 replicate cages (*n* = 7)

^2^Data represent the means of 21 replicate cages (*n* = 21)

^3^Data represent the means of 28 replicate cages (*n* = 28)

^4^Linear effects of added Fe levels

^A,B^Means with different superscripts between Fe sources differ (*P* < 0.05)

^a,b^Means with different superscripts between added Fe levels differ (*P* < 0.05)

*Different (*P* < 0.05) from all Fe supplemental groups

### mRNA expression levels

The data were shown in Table 8. Compared with the control, dietary Fe supplementation had no effect (*P* > 0.05) on *CAT* and *COX* mRNA expression levels in heart, kidney, and liver, as well as *SDH* mRNA in heart, but increased (*P* < 0.05) mRNA expression levels of *SDH* in kidney and liver. The mRNA expression levels of heart *CAT* and *SDH* in kidney and liver were affected (*P* < 0.03) by the Fe source. Compared with the FeSO_4_∙7H_2_O, feed grade or food grade NaFeEDTA up-regulated (*P* < 0.002) the heart *CAT* mRNA expression level. Chicks fed with the diet supplemented with feed grade NaFeEDTA had a higher (*P* = 0.0004) heart *CAT* mRNA expression level than those fed with the diet supplemented with Fe-Prot ES, and no differences (*P* > 0.05) were observed between FeSO_4_∙7H_2_O and Fe-Prot ES, Fe-Prot ES and food grade NaFeEDTA, as well as between feed grade and food grade NaFeEDTA. Compared to the FeSO_4_∙7H_2_O, the 3 organic Fe sources increased (*P* < 0.02) *SDH* mRNA expression levels in liver and kidney. No differences (*P* > 0.34) were observed in *SDH* mRNA expression levels in liver and kidney among the 3 organic Fe sources. In addition, mRNA expression levels of *SDH* in liver and kidney and liver *COX* were affected (*P* < 0.05) by added Fe level, but only *SDH* mRNA expression levels in kidney and liver increased linearly (*P* ≤ 0.0009); liver *COX* mRNA level did not increase linearly (*P* > 0.05) with increasing added Fe levels. No effects (*P* > 0.07) were found regarding the interaction between Fe source and added Fe level on all of the above indices as well as Fe source or added Fe level on other indices.

**Table 8:** Effects of dietary Fe on mRNA levels of tissue Fe-containing enzymes of broilers on d 21

Fe source	Added Fe, mg/kg	*CAT* mRNA^5^, RQ			*SDH* mRNA^5^, RQ			*COX* mRNA^5^, RQ		
		Heart	Kidney	Liver	Heart	Kidney	Liver	Heart	Kidney	Liver
Control^1^	0	1.01	1.02	1.01	1.01	1.01*	1.03*	1.01	1.00	1.01
FeSO_4_∙7H_2_O^1^	20	1.05	0.97	0.98	1.02	1.05	1.05	0.88	0.89	1.01
	40	0.96	1.02	1.21	0.91	1.10	1.17	1.09	1.08	0.99
	60	0.95	0.95	1.19	1.02	1.19	1.30	0.84	0.94	1.16
Fe-Prot ES^1^	20	1.13	0.93	0.99	0.98	1.16	1.23	0.91	0.93	1.08
	40	1.13	0.90	1.18	0.98	1.31	1.36	1.00	0.94	1.08
	60	1.13	0.92	0.91	0.97	1.42	1.46	0.94	0.99	1.21
Feed grade NaFeEDTA^1^	20	1.54	1.03	1.11	1.04	1.19	1.20	1.07	0.94	1.08
	40	1.58	0.95	1.19	0.85	1.30	1.47	0.94	1.02	1.08
	60	1.48	1.11	1.02	0.94	1.44	1.51	1.01	1.12	1.15
Food grade NaFeEDTA^1^	20	1.26	0.98	1.03	0.97	1.26	1.39	0.94	0.90	0.94
	40	1.27	0.98	1.15	0.93	1.23	1.37	0.96	0.86	1.07
	60	1.49	0.91	0.95	1.00	1.35	1.51	0.96	0.85	1.23
Pooled SE		0.13	0.07	0.11	0.07	0.08	0.09	0.1	0.07	0.09
Fe source^2^	FeSO_4_∙7H_2_O	0.99^C^	0.98	1.13	0.98	1.11^B^	1.17^B^	0.94	0.97	1.05
	Fe-Prot ES	1.13^BC^	0.92	1.03	0.98	1.30^A^	1.35^A^	0.95	0.95	1.13
	Feed grade NaFeEDTA	1.53^A^	1.03	1.11	0.95	1.31^A^	1.39^A^	1.01	1.03	1.10
	Food grade NaFeEDTA	1.34^AB^	0.96	1.04	0.97	1.28^A^	1.42^A^	0.95	0.87	1.08
Pooled SE		0.08	0.04	0.07	0.04	0.06	0.05	0.05	0.04	0.06
Added Fe level^3^, mg/kg	20	1.25	0.98	1.03	1.00	1.16^b^	1.22^b^	0.95	0.91	1.03^b^
	40	1.24	0.96	1.18	0.92	1.24^ab^	1.34^ab^	1.00	0.97	1.06^ab^
	60	1.26	0.97	1.01	98	1.35 ^a^	1.45^a^	0.94	0.97	1.18^a^
Pooled SE		0.07	0.03	0.06	0.04	0.04	0.05	0.05	0.04	0.05
*P*-value	Fe source	< 0.0001	0.2679	0.6672	0.9303	0.0143	0.0048	0.8188	0.0792	0.8131
	Added Fe level	0.9562	0.9508	0.0954	0.2172	0.0091	0.0027	0.6265	0.4327	0.0423
	Fe Source × added Fe level	0.8794	0.6658	0.8536	0.8476	0.9340	0.8465	0.6108	0.5054	0.9464
	Linear effect^4^	–	–	–	–	0.0003	0.0001	–	–	0.0596

*CAT* Catalase, *SDH* Succinate dehydrogenase, *COX* Cytochrome c oxidase

^1^Data represent the means of 7 replicate cages (*n* = 7)

^2^Data represent the means of 21 replicate cages (*n* = 21)

^3^Data represent the means of 28 replicate cages (*n* = 28)

^4^Linear effects of added Fe levels

^5^The *CAT*, *SDH* or *COX* mRNA abundances were calculated as the relative quantity (RQ) of the *CAT*, *SDH* or *COX* mRNA to the geometric mean of β-actin and *GAPDH* mRNA; RQ = 2^-△△Ct^ (Ct = threshold cycle)

^A–C^Means with different superscripts between Fe sources differ (*P* < 0.05)

^a,b^Means with different superscripts between added Fe levels differ (*P* < 0.05)

*Different (*P* < 0.05) from all Fe supplemental groups

### Protein expression levels

**Fig. 1:**
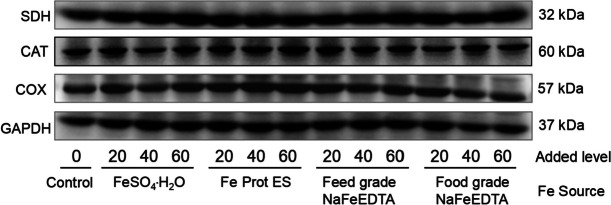
Representative Western blot images demonstrating the protein expression levels of SDH, CAT and COX in liver of broilers at 21 days of age. CAT = catalase; SDH = succinate dehydrogenase; COX = cytochrome c oxidase

The data were shown in Fig. 1 and Table 9. Compared to the control, dietary Fe supplementation had no effect (*P* > 0.05) on protein expression levels of CAT and SDH in liver, but enhanced (*P* < 0.05) the COX protein expression level in liver. Meanwhile, Fe source, added Fe level, and their interaction did not affect (*P* > 0.14) protein expression levels of the above 3 Fe-containing enzymes in liver.

**Table 9:** Effects of dietary Fe on protein levels of liver Fe-containing enzymes of broilers on d 21

Fe source	Added Fe, mg/kg	CAT^4^, RQ	SDH^4^, RQ	COX^4^, RQ
Control^1^	0	0.85	0.91	0.76*
FeSO_4_∙7H_2_O^1^	20	0.88	0.91	0.82
	40	0.96	0.90	0.92
	60	0.98	0.88	0.94
Fe-Prot ES^1^	20	0.83	0.84	0.93
	40	1.08	0.91	1.01
	60	0.83	0.77	0.92
Feed grade NaFeEDTA^1^	20	0.98	0.91	0.95
	40	0.94	0.93	0.86
	60	0.90	1.02	0.90
Food grade NaFeEDTA^1^	20	0.89	1.08	1.03
	40	0.91	0.99	1.02
	60	0.83	0.94	0.93
Pooled SE		0.12	0.09	0.08
Fe source^2^	FeSO_4_∙7H_2_O	0.94	0.90	0.90
	Fe-Prot ES	0.91	0.84	0.95
	Feed grade NaFeEDTA	0.94	0.95	0.88
	Food grade NaFeEDTA	0.88	1.00	0.99
Pooled SE		0.07	0.05	0.05
Added Fe level^3^, mg/kg	20	0.90	0.93	0.93
	40	0.97	0.93	0.95
	60	0.89	0.90	0.90
Pooled SE		0.06	0.05	0.04
*P*-value	Fe source	0.9043	0.1428	0.3103
	Added Fe level	0.5165	0.8610	0.6673
	Fe Source × added Fe level	0.8383	0.7942	0.7392

*CAT* Catalase, *SDH* Succinate dehydrogenase, *COX* Cytochrome c oxidase

^1^Data represent the means of 7 replicate cages (*n* = 7)

^2^Data represent the means of 21 replicate cages (*n* = 21)

^3^Data represent the means of 28 replicate cages (*n* = 28)

^4^The protein expression levels were calculated as the relative quantities (RQ) of the target gene protein to the GAPDH protein

*Different (*P* < 0.05) from all Fe supplemental groups

### Estimation of relative bioavailability values

Multiple linear regressions were conducted between the related dependent variables and daily dietary analyzed Fe intakes of different Fe sources during the experimental period (Table 10). Significant (*P* < 0.04) multiple linear regression relationships were observed in PI and TS in plasma, liver Fe content, SDH activities in liver, heart and kidney, CAT and COX activities in liver, and *SDH* mRNA expression levels in liver and kidney. Therefore, based on the above multiple linear regression equations in Table 10, the bioavailability values of 3 organic Fe sources relative to FeSO_4_∙7H_2_O were estimated (Table 11). Differences (*P* ≤ 0.03) in slopes among Fe sources were detected in PI, Fe content, and CAT activity in liver, SDH activities in heart and kidney, and *SDH* mRNA expression levels in liver and kidney. No differences (*P* ≥ 0.15) in slopes among Fe sources were detected in plasma TS, as well as SDH and COX activities in liver. When the response to reagent grade FeSO_4_∙7H_2_O was set to 100%, the relative bioavailabilities of feed grade Fe-Prot, feed grade NaFeEDTA, and food grade NaFeEDTA were 95% (*P* > 0.60), 106% (*P* > 0.43) and 120% (*P* < 0.02) (PI); 159% (*P* < 0.01), 179% (*P* < 0.0005), and 187% (*P* < 0.0002) (liver Fe content); 111% (*P* > 0.55), 133% (*P* > 0.09), and 144% (*P* < 0.03) (heart SDH activity); 138% (*P* > 0.12), 179% (*P* < 0.001), and 185% (*P* < 0.0007) (kidney SDH activity); 155% (*P* < 0.005), 156% (*P* < 0.004), and 194% (*P* < 0.0001) (liver CAT activity); 147% (*P* < 0.02), 160% (*P* < 0.002), and 169% (*P* < 0.0006) (liver *SDH* mRNA expression level); 170% (*P* < 0.003), 174% (*P* < 0.002), and 160% (*P* < 0.002) (kidney *SDH* mRNA expression level). The average relative bioavailabilities of Fe-Prot ES, feed grade NaFeEDTA, and food grade NaFeEDTA relative to FeSO_4_∙7H_2_O (100%) in broiler chicks were 139%, 155%, and 166%, respectively.

**Table 10:** Multiple linear regressions of dependent variables on daily dietary analyzed Fe intake^1^

Dependent variable	Regression equation^2^	*R*^2^	*P*-value
PI	*Y* = 0.6261 + 0.1137*X*_1_ + 0.1084*X*_2_ + 0.1216*X*_3_ + 0.1368*X*_4_	0.42	< 0.0001
TS	*Y* = 14.1258 + 4.5054*X*_1_ + 4.2214*X*_2_ + 4.562*X*_3_ + 4.9182*X*_4_	0.35	< 0.0001
Liver Fe	*Y* = 67.092 + 5.821*X*_1_ + 9.228*X*_2_ + 10.414*X*_3_ + 10.883*X*_4_	0.34	< 0.0001
Liver SDH activity	*Y* = 2.0216 + 0.1080*X*_1_ + 0.1310*X*_2_ + 0.1365*X*_3_ + 0.1465*X*_4_	0.12	0.0329
Heart SDH activity	*Y* = 2.5816 + 0.2538*X*_1_ + 0.2834*X*_2_ + 0.3371*X*_3_ + 0.3653*X*_4_	0.22	0.001
Kidney SDH activity	*Y* = 3.4912 + 0.2365*X*_1_ + 0.3272*X*_2_ + 0.4243*X*_3_ + 0.4378*X*_4_	0.28	< 0.0001
Liver CAT activity	*Y* = 14.9025 + 0.3275*X*_1_ + 0.5077*X*_2_ + 0.5121*X*_3_ + 0.6353*X*_4_	0.37	< 0.0001
Liver COX activity	*Y* = 20.679 + 2.236*X*_1_ + 2.450*X*_2_ + 2.410*X*_3_ + 2.572*X*_4_	0.25	< 0.0001
Liver *SDH* mRNA	*Y* = 0.7122 + 0.0604*X*_1_ + 0.0865*X*_2_ + 0.09490*X*_3_ + 0.0978*X*_4_	0.26	< 0.0001
Kidney *SDH* mRNA	*Y* = 0.8306 + 0.0537*X*_1_ + 0.0924*X*_2_ + 0.0943*X*_3_ + 0.0882*X*_4_	0.29	< 0.0001

*PI* Plasma iron, *TS* Transferrin saturation, *CAT* Catalase, *SDH* Succinate dehydrogenase, *COX* Cytochrome c oxidase

^1^Daily dietary analyzed Fe intake = average daily feed intake during 1–21 days of age times the dietary analyzed Fe content for each respective Fe source. Regression analyses of PI, TS in plasma, liver Fe contents, tissue CAT, COX and SDH activities, and *SDH* mRNA expression levels were based on cage averages with 21 cages (3 chicks killed/cage) per Fe source

^2^*Y* is the PI (μg/mL), TS in plasma (%), tissue SDH and CAT enzyme activities (U/mg protein), liver Fe (μg/g, fresh basis), and *SDH* mRNA expression levels (RQ) in liver and kidney; *X*_1_ is the daily dietary analyzed Fe intake (mg) for FeSO_4_∙7H_2_O; *X*_2_ is the daily dietary analyzed Fe intake (mg) for Fe-Prot ES; *X*_3_ is the daily dietary analyzed Fe intake (mg) for feed grade NaFeEDTA; *X*_4_ is the daily dietary analyzed Fe intake (mg) for food grade NaFeEDTA

**Table 11:** Estimation of relative bioavailability values (RBV) of the 3 organic Fe sources^1^

	Regression coefficient, slope (mean ± SE)				RBV, % (mean ± SE)				
Dependent variable	FeSO_4_∙7H_2_O	Fe-Prot ES	Feed grade NaFeEDTA	Food grade NaFeEDTA	FeSO_4_∙7H_2_O	Fe-Prot ES	Feed grade NaFeEDTA	Food grade NaFeEDTA	*P*-value^2^
PI	0.114 ± 0.019^B^	0.108 ± 0.019^B^	0.122 ± 0.018^AB^	0.137 ± 0.019^A^	100	95 ± 9	106 ± 8	120 ± 8	0.02
TS	4.505 ± 0.845	4.221 ± 0.801	4.562 ± 0.773	4.918 ± 0.806	100	94 ± 15	101 ± 14	109 ± 14	0.34
Liver Fe	5.821 ± 2.513^B^	9.228 ± 2.445^A^	10.414 ± 2.387^A^	10.883 ± 2.506^A^	100	159 ± 24	179 ± 26	187 ± 26	< 0.0001
Liver SDH activity	0.108 ± 0.051	0.131 ± 0.049	0.137 ± 0.048	0.147 ± 0.050	100	121 ± 23	127 ± 23	136 ± 24	0.15
Heart SDH activity	0.254 ± 0.095^B^	0.283 ± 0.091^AB^	0.337 ± 0.091^AB^	0.365 ± 0.093^A^	100	111 ± 19	133 ± 19	144 ± 20	0.03
Kidney SDH activity	0.237 ± 0.111^B^	0.327 ± 0.109^AB^	0.424 ± 0.106^A^	0.438 ± 0.111^A^	100	138 ± 24	179 ± 28	185 ± 28	0.0007
Liver CAT activity	0.328 ± 0.123^C^	0.508 ± 0.119^B^	0.512 ± 0.119^AB^	0.635 ± 0.122^A^	100	155 ± 21	156 ± 21	194 ± 23	< 0.0001
Liver COX activity	2.236 ± 0.533	2.450 ± 0.519	2.410 ± 0.514	2.572 ± 0.522	100	112 ± 12	108 ± 12	115 ± 12	0.23
Liver *SDH* mRNA	0.073 ± 0.027^B^	0.107 ± 0.027^A^	0.117 ± 0.027^A^	0.123 ± 0.027^A^	100	147 ± 20	160 ± 21	169 ± 21	< 0.0001
Kidney *SDH* mRNA	0.054 ± 0.024^B^	0.092 ± 0.023^A^	0.094 ± 0.023^A^	0.088 ± 0.024^A^	100	170 ± 25	174 ± 26	160 ± 25	< 0.0001

*PI* Plasma iron, *TS* Transferrin saturation, *CAT* Catalase, *SDH* Succinate dehydrogenase, *COX* Cytochrome C oxidase

^1^Based on multiple linear regressions of the above dependent variables on daily dietary analyzed Fe intake. Daily dietary analyzed Fe intake = average daily feed intake during 1–21 days of age times the dietary analyzed Fe content for each respective Fe source. Regression analyses of the above dependent variables were based on cage averages with 21 cages (3 chicks killed/cage) per Fe source

^2^*P*-value for the difference in slopes among Fe sources

^A–C^Means with different superscripts within the same row differ (*P* < 0.05)

When the relative bioavailabilities were estimated based on PI, the slope was greater (*P* < 0.05) for food grade NaFeEDTA than for FeSO_4_∙7H_2_O or Fe-Prot ES; no differences (*P* > 0.05) were found between feed grade NaFeEDTA and each of the other 3 Fe sources as well as between FeSO_4_∙7H_2_O and Fe-Prot ES. When the relative bioavailabilities were estimated based on liver Fe content and *SDH* mRNA expression levels in liver and kidney, the slopes were greater (*P* < 0.05) for Fe-Prot ES, feed grade NaFeEDTA, and food grade NaFeEDTA than for FeSO_4_∙7H_2_O; no differences (*P* > 0.05) were found among the 3 organic Fe sources. When the relative bioavailabilities were estimated based on heart SDH activity, the slope was only greater (*P* < 0.05) for food grade NaFeEDTA than for FeSO_4_∙7H_2_O with no differences (*P* > 0.05) among other Fe sources. When the relative bioavailabilities were estimated based on kidney SDH activity, the slope was greater (*P* < 0.05) for feed grade or food grade NaFeEDTA than for FeSO_4_∙7H_2_O; no differences (*P* > 0.05) were observed between Fe-Prot ES and each of the other Fe sources as well as between feed grade NaFeEDTA and food grade NaFeEDTA. When the relative bioavailabilities were estimated based on liver CAT activity, the slopes were greater (*P* < 0.05) for Fe-Prot ES, feed grade, or food grade NaFeEDTA than for FeSO_4_∙7H_2_O; slopes were also greater (*P* < 0.05) for food grade NaFeEDTA than for Fe-Prot ES and no differences (*P* > 0.05) were observed between Fe-Prot ES and feed grade NaFeEDTA as well as between feed grade NaFeEDTA and food grade NaFeEDTA.

## Discussion

In the present study, we found that the Q_f_ values of both feed grade NaFeEDTA and food grade NaFeEDTA were as high as 2.07 × 10^8^ and 3.31 × 10^8^, respectively. These values were much higher than that (8,590) of the Fe-Prot ES used in the current and previous studies [12]. Such high values belong to super extremely strong chelation strengths according to the Q_f_ value classification of Holwerda et al. for organic trace elements [31]. Furthermore, the average bioavailabilities of both feed grade NaFeEDTA and food grade NaFeEDTA relative to FeSO_4_·7H_2_O (100%) were 155% and 166%, respectively; however, the average bioavailability of Fe-Prot ES relative to FeSO_4_·7H_2_O (100%) was 139%, indicating that the NaFeEDTA sources with the greatest Q_f_ values achieved the highest Fe bioavailabilities in broilers. The above findings have supported the proposed hypothesis, and have been not reported in broilers and all of other agricultural animals before. New insight and scientific basis were provided for the promotion and application of the highly bioavailable NaFeEDTA as a new feed Fe additive to minimize an excessive Fe addition to diets and manure Fe excretion to the environment in the poultry production.

The bioavailability of trace minerals refers to the proportion of the element ingested from a specific source that is absorbed, transported to its action site, and converted into physiologically active forms that can serve the animal metabolism [32]. Therefore, the selection of sensitive criteria is crucial for the evaluation of the bioavailability of trace elements [24]. Researchers have performed a series of experiments to estimate the bioavailabilities of different forms of organic Fe relative to the traditional inorganic Fe sulfate in animals, but the results were inconsistent [13, 33, 34]. These disparities in organic Fe bioavailabilities might depend upon a variety of different factors, especially the chemical characteristics of different Fe sources which are considered important for predicting the bioavailabilities of complexed or chelated metals [12]. Many studies from our laboratory have led to the valuable new finding, indicating that the complex or chelation strengths (Q_f_ value) of organic Mn, Zn, Fe, or Cu sources are closely related to their bioavailabilities in both broilers and lactating cows [12–14, 24, 35–40]. Regarding organic Fe sources, the greater the Q_f_ values, the greater their relative bioavailabilities in broilers [12, 13]. Therefore, the results of the present study further support and confirm our previous results. In addition, the Fe-Prot ES used in the present study was the same as that used in our previous study of Zhang et al. [12]. The bioavailabilities of the Fe-Prot ES relative to FeSO_4_∙7H_2_O (100%) as estimated based on the same indices (*SDH* mRNA expression levels in the liver and kidney of broilers on d 21) averaged 159% in the present study and 174% in our previous study [12], indicating that these results are repeatable and reliable.

Growth performance indices are generally unresponsive to the addition of many mineral elements to practical diets [24, 41]. The results of the present study are consistent with those of previous researches in broilers [12, 33, 42], indicating that the growth performance indicators are not suitable for the evaluation of bioavailabilities of Fe sources in broilers fed with a conventional practical diet.

Hematological indices have been proved to be the responsive criteria to determine Fe bioavailability [43–46]. For instance, when FeSO_4_∙7H_2_O was set to 100%, the relative Fe bioavailability of Fe glycinate was about 90% based on Hb concentration in rats [47]. Using a purified casein-dextrose basal diet containing 4.56 mg Fe/kg, Ma et al. [13], demonstrated that blood Hb concentration and total body Hb Fe were sensitive indices in reflecting differences in bioavailability among different Fe sources; the Fe proteinate with the moderate chelation strength (Q_f_ = 43.6) was significantly more available (116%) to broilers than inorganic FeSO_4_∙7H_2_O (100%) in enhancing Hb concentration and total body Hb Fe. However, Zhang et al. [12], used a practical corn-soybean meal basal diet containing 55.8 mg Fe/kg, and found that the hematological indices (i.e., Hb, Hct, PI, TIBC, and TS) were not sufficiently sensitive indicators for evaluating the bioavailabilities of Fe sources, majorly due to the higher background Fe content in the practical corn-soybean meal basal diet. The results of the present study, where a similar practical corn-soybean meal basal diet containing 67.9 mg Fe/kg was employed, were partially consistent with the results of Zhang et al. regarding Hb, Hct, or TIBC; however, PI and TS were found to increase linearly with increasing added Fe levels in diets [12]. Furthermore, the bioavailability of food grade NaFeEDTA was estimated to be significantly higher than the bioavailability of FeSO_4_∙7H_2_O and Fe-Prot ES based on PI, indicating that PI could be a sensitive criterion for evaluating the bioavailabilities of Fe sources in broilers.

Tissue Fe accumulation has been considered a sensitive criterion for evaluating Fe bioavailability [48]. As liver is the main site of the body Fe storage and metabolism in the body, liver Fe content has been used as the evaluating criterion to compare the bioavailabilities of different Fe sources. It was reported that Fe contents in liver and kidney of broiler chicks fed with corn-soybean meal diets were increased linearly by added Fe level; further, the relative bioavailability of Fe methionine as estimated based on liver Fe content was 88.3% if FeSO_4_∙7H_2_O was set to 100% [49]. Yu et al. [50], demonstrated that the Fe-amino acid complex was more effective than FeSO_4_ in promoting liver Fe accumulation in weanling pigs. It is worth noting that in a recent study, compared with FeSO_4_, dietary supplementation with NaFeEDTA increased Fe contents in mouse liver, kidney, and blood, and decreased the bioavailability of the heavy metal lead (Pb) [51]. In the current study, Fe contents in liver and heart were affected by Fe source, but only liver Fe content exhibited a significantly linear increase as dietary added Fe levels increased, indicating that the liver Fe content could be a sensitive criterion for estimating the bioavailabilities of Fe sources in broilers.

The Fe is an indispensable cofactor of many enzymes, such as CAT, SDH, and COX, and the activities of these Fe-containing enzymes play a crucial role in maintaining animal physiological homeostasis [52]. CAT is an antioxidant enzyme that prevents cells from the oxidative damage by degrading hydrogen peroxide to water and oxygen [53]. The SDH and COX are two respiratory enzymes, localized in the mitochondrial membrane, and they are essential for mitochondrial function [54]. It has been widely accepted that Fe source and Fe level can affect the activities of Fe-containing enzymes [55, 56]. Liver, heart, and kidney are specific target tissues that are particularly rich in these Fe-containing enzymes. Ma et al. [57], reported that the activities of SDH, CAT, and COX in the liver, and the activity of SDH in the heart of broilers fed with a corn-soybean meal basal diet supplemented with FeSO_4_∙7H_2_O increased quadratically with increasing dietary added Fe levels. Feng et al. [58], reported that liver SDH activity in weaned piglets increased linearly with increasing dietary Fe levels. However, Zhang et al. [12], found that CAT activities in the above 3 tissues, as well as SDH activity in heart of broilers at 21 days of age were not affected by Fe source and added Fe level. In the present study, CAT activity in liver and SDH activity in kidney of broilers at 21 days of age were affected by Fe source, and increased linearly with increasing supplemental Fe levels. Meanwhile, heart SDH activity also increased linearly with increasing added Fe levels, and there was a significant difference in Fe bioavailability between FeSO_4_∙7H_2_O and food grade NaFeEDTA. These results imply that the above 3 indices could be specifically sensitive functional criteria for estimating the bioavailabilities of Fe sources in broilers. The above inconsistency between the present study and previous studies might be related to the differences in Fe source, added Fe level, and broiler sources.

Previous studies have demonstrated that the mRNA expression levels of key enzymes or functional proteins in target tissues of animals are highly sensitive to the changes in dietary trace element contents [24, 41, 54]. For instance, *SDH* mRNA expression levels in liver and kidney of broilers on d 21 increased linearly with increasing added Fe levels; these were sensitive criteria for estimating the relative bioavailabilities of organic Fe sources with different chelation strengths [12]. Herein, mRNA expression levels of Fe-containing enzymes (SDH, CAT, and COX) in various tissues (heart, kidney and liver) of broiler chicks at 21 days of age were also determined; the results showed that only *SDH* mRNA expression levels in liver and kidney were influenced by both Fe source and added Fe level, as well as increased linearly with increasing dietary added Fe levels. Thus, these are particularly sensitive functional indices for evaluating the bioavailabilities of Fe sources in broilers, which is completely consistent with the results of Zhang et al. [12] from our laboratory. In addition, protein expression levels of SDH, CAT, and COX in the liver of broilers on d 21 were also assessed. The results showed that these indices were not affected by either Fe source or added Fe level, indicating that protein expression levels of the above Fe-containing enzymes in liver were not sensitive indices for assessing the bioavailabilities of Fe sources for broilers. As no specific antibodies for detecting protein expressions of the above Fe-containing enzymes in chicks are available now, other nonspecific antibodies (such as anti-rabbit antibody and anti-mouse antibody) had to be used to assess the above protein expressions. Furthermore, because protein expressions are affected by more complicated factors, and are usually delayed for a certain time after the corresponding mRNA expressions, it would be reasonable to assume that the mRNA expression levels of *SDH* in tissues are more sensitive than the protein expression levels detected in the present study.

As mentioned above, the bioavailabilities of organic trace elements in animals are largely determined by their complex or chelation strengths (Q_f_ values). Previous studies in our laboratory using broilers as model animals have indicated that the organic Mn or Zn sources with moderate complex or chelation strengths (Q_f_ values = 10–100) displayed the highest relative bioavailabilities [24, 35, 37]. Further, the organic Mn, Zn, or Cu sources with weak complex strengths (Q_f_ values < 10) were comparable to their inorganic forms [24, 35, 37], while the bioavailability of the organic Zn with near extremely strong chelation strength (Q_f_ value = 944) was significantly lower than that of the inorganic ZnSO_4_ [24, 35, 37]. However, regarding organic Fe sources, the obtained results were inconsistent with the above conclusions. The results of the present study and our previous research in broilers consistently demonstrated that the greater the Q_f_ values of organic Fe sources, the higher their bioavailabilities relative to FeSO_4_∙7H_2_O [12]; the NaFeEDTA sources with the greatest Q_f_ values displayed the highest Fe bioavailabilities. The reasons for the above discrepancies might be related to the different chemical characteristic and absorptive mechanism of Fe in the small intestine. Compared to other essential trace elements such as Mn, Zn, and Cu, Fe is relatively active and more easily affected by complex factors (such as pH, Ca, phytate, and fibers) in the gastrointestinal tract. To improve Fe absorption and utilization, it is therefore necessary to use the strongest ligand such as EDTA to protect Fe against the interferences from these factors in the gastrointestinal tract. Therefore, the organic NaFeEDTA sources with the strongest chelation strengths could better resist to the above complex interferences. More Fe in the form of NaFeEDTA arrived at the absorptive site on the brush edge surface of the small intestine, where it might be dissociated and absorbed in the form of the ionized Fe for target tissue utilization; thus, the NaFeEDTA sources were more available to broilers. However, as more Zn from the organic Zn with near extremely strong chelation strength arrived at the absorptive site, the binding strength of this chelated Zn might be stronger than the binding strength of Zn transporters, leading to a lesser Zn bioavailability. Additionally, even if this chelated Zn could be absorbed in the form of the intact chelated molecular Zn, such absorbed Zn might be not better released from the intact chelated molecule for the target tissue utilization because of its strong chelation strength, and thus, it would be less available to broilers. Further research is required to identify the Fe absorption mechanisms when provided in the form of NaFeEDTA in the small intestine of broilers as well as its metabolic utilization and mechanisms in the target tissue of broilers to confirm the above speculations.

## Conclusion

The present study showed that PI, liver Fe content, SDH activities in kidney and heart, liver CAT activity, and *SDH* mRNA expression levels in liver and kidney of 21-day-old broilers were sensitive indices to evaluate the bioavailabilities of Fe sources in broilers chicks fed with a corn-soybean meal diet during 1–21 days of age. The bioavailabilities of organic Fe sources relative to FeSO_4_∙7H_2_O (100%) were closely related to their Q_f_ values, and the NaFeEDTA sources with the greatest Q_f_ values (2.07 × 10^8^–3.31 × 10^8^) displayed the highest Fe bioavailabilities (155%–166%).

## Data Availability

The datasets used and/or analyzed during the current study are available from the corresponding author on reasonable request.

## Abbreviations

AA: Arbor Acres
ADFI: Average daily feed intake
ADG: Average daily gain
BCA: Bicinchoninic acid
Ca: Calcium
Ca(CH_3_COO)_2_·H_2_O: Calcium acetate monohydrate
CAT: Catalase
COX: Cytochrome c oxidase
E_1/2_: Half-wave potential
Fe: Iron
Fe-Prot ES: Iron proteinate with an extremely strong chelation strength
FeSO_4_·7H_2_O: Ferrous sulfate
F/G: Feed to gain ratio
GAPDH: Glyceraldehyde-3-phosphate dehydrogenase
Hb: Hemoglobin
Hct: Hematocrit
NaFeEDTA: Sodium iron ethylenediaminetetraacetate
Na_2_S_2_O_3_: Sodium thiosulfate
PI: Plasma iron
Q_f_: Quotient of formation
RT-qPCR: Quantitative real-time polymerase chain reaction
SDH: Succinate dehydrogenase
TIBC: Total iron binding capacity
TS: Transferrin saturation
